# Extreme spatial confinement and high site fidelity in a crevice-dwelling lizard with a minimal home range

**DOI:** 10.1186/s40850-025-00253-z

**Published:** 2025-12-26

**Authors:** R. Isaac Rojas-González, Isaac E. Diaz-Ortega

**Affiliations:** 1 Dirección de Investigación Pesquera en el Atlántico, Instituto Mexicano de Investigación en Pesca y Acuacultura Sustentables (IMIPAS), Av. México 190, Coyoacán, Mexico City, 04110 Mexico; 2 Laboratorio de Ecología Evolutiva de Anfibios y Reptiles, Carrera de Biología, Facultad de Estudios Superiores Iztacala, Universidad Nacional Autónoma de México (UNAM), Av. de los Barrios 1, Los Reyes Ixtacala, Tlalnepantla de Baz, State of Mexico 54090 Mexico

**Keywords:** Bayesian modelling, Secretive lizards, Spatial behaviour, Thermal ecology, Thigmothermy, *Xenosaurus platyceps*

## Abstract

**Background:**

Body size has traditionally been regarded as a key predictor of home-range extent. However, it remains unclear whether habitat specialisation can alter the expected allometric relationship between body size and home range. Crevice-dwelling lizards of the genus *Xenosaurus* provide an excellent system for addressing this question due to their extremely restricted habitat use. Using Bayesian generalised linear models—which allow explicit comparison of alternative hypotheses—we focused on (I) characterising the spatial behaviour of *X. platyceps*, (II) testing whether the home range of *X. platyceps* deviates from allometric expectations within a comparative dataset of 100 lizard species, and (III) evaluating whether the thermal environment helps explain its reduced movement.

**Results:**

We report one of the smallest and most temporally consistent home ranges documented for an adult lizard. It was observed in a female *X. platyceps* that remained in the same crevice for more than 43 months, with an estimated minimum home range of 0.014 m^2^. Bayesian allometric analyses showed that the observed value was over two orders of magnitude smaller than predictions based on body-size allometry. Thermal modelling revealed no differences between body, ambient and crevice temperatures. Moreover, body temperature was best explained by crevice temperature. These results indicate a thermally homogeneous microhabitat and thermoconforming behaviour. This pattern may help explain the reduced movement observed in *X. platyceps.*

**Conclusions:**

Our findings suggest that, in species with extreme habitat specialisation, home-range extent may be better explained by environmental stability and foraging strategy than by the classical body-size relationship. These results highlight the potential for tight microhabitat coupling to decouple spatial use from allometric expectations. Moreover, because microhabitat specialists are particularly vulnerable to habitat fragmentation and refuge loss, it is essential to consider spatial use patterns when assessing conservation status and designing management strategies.

**Supplementary information:**

The online version contains supplementary material available at 10.1186/s40850-025-00253-z.

## Background

Home range is the spatial area in which an animal carries out its regular activities [1–3]. It is therefore a key metric for understanding behavioural ecology and informing conservation strategies [4, 5]. Although home range has been linked to environmental conditions, resource availability and social interactions [5–7], intrinsic species-level factors—such as habitat specialisation—may also strongly influence its extent.

In lizards, home-range size is often associated with body size and foraging mode [5, 6, 8]. Larger individuals are expected to use broader areas because they have higher energetic demands and require more resources to meet them [6]. Classical predictions suggest that large-bodied individuals and active foragers should maintain broader home ranges, whereas smaller species and sit-and-wait predators typically occupy more restricted areas [9]. However, in species with extreme habitat specialisation, space use may be driven mainly by strong site fidelity rather than by movement patterns associated with body size or foraging behaviour.

Species’ thermal ecology can also influence movement patterns [10, 11]. In thermoconforming species, movement may be reduced because their physiological requirements do not depend on maintaining a narrow optimal temperature range [12]. Moreover, when the habitat provides thermally stable conditions, movement can decrease, reducing the energetic costs associated with locomotion [11, 13]. This effect may be especially pronounced in thigmothermic species, which obtain their thermal requirements directly from the substrate. Collectively, thermoconformity and thigmothermy may constrain home-range extent, producing a decoupling with size-based allometric predictions.

*Xenosaurus platyceps* (family Xenosauridae) is a secretive lizard with a distribution restricted to isolated localities in Mexico [14, 15]. It is an extreme crevice specialist, typically found in rocky outcrops or tree bark fissures. Species in this genus exhibit reduced movement, a sit-and-wait foraging strategy, and thermoconformist behaviour [16–18]. These traits make *X. platyceps* an ideal model to assess potential mismatches between observed home-range extent and allometric predictions in extremely specialised species.

In this study, we applied Bayesian statistical tools to evaluate whether the space use of *X. platyceps* aligns with ecological predictions based on body size. Specifically, we aimed to (I) document the spatial behaviour of individuals monitored over 3.5 years in a spatially restricted habitat; (II) assess whether the observed home range matches expectations for similarly sized lizards [6, 19]; and (III) explore the thermal context of refuges and individuals as a potential explanatory factor for such restricted spatial behaviour. We considered that extreme habitat specialisation may lead to a decoupling from the positive allometric relationship between home-range extent and body size [7, 6]. Specifically, we anticipated that the home range of *X. platyceps* would be smaller than expected from body-size allometry, even when compared with smaller species. We also expected that the thermal environment would facilitate reduced movement and prolonged refuge use.

## Methods

**Fig. 1:**
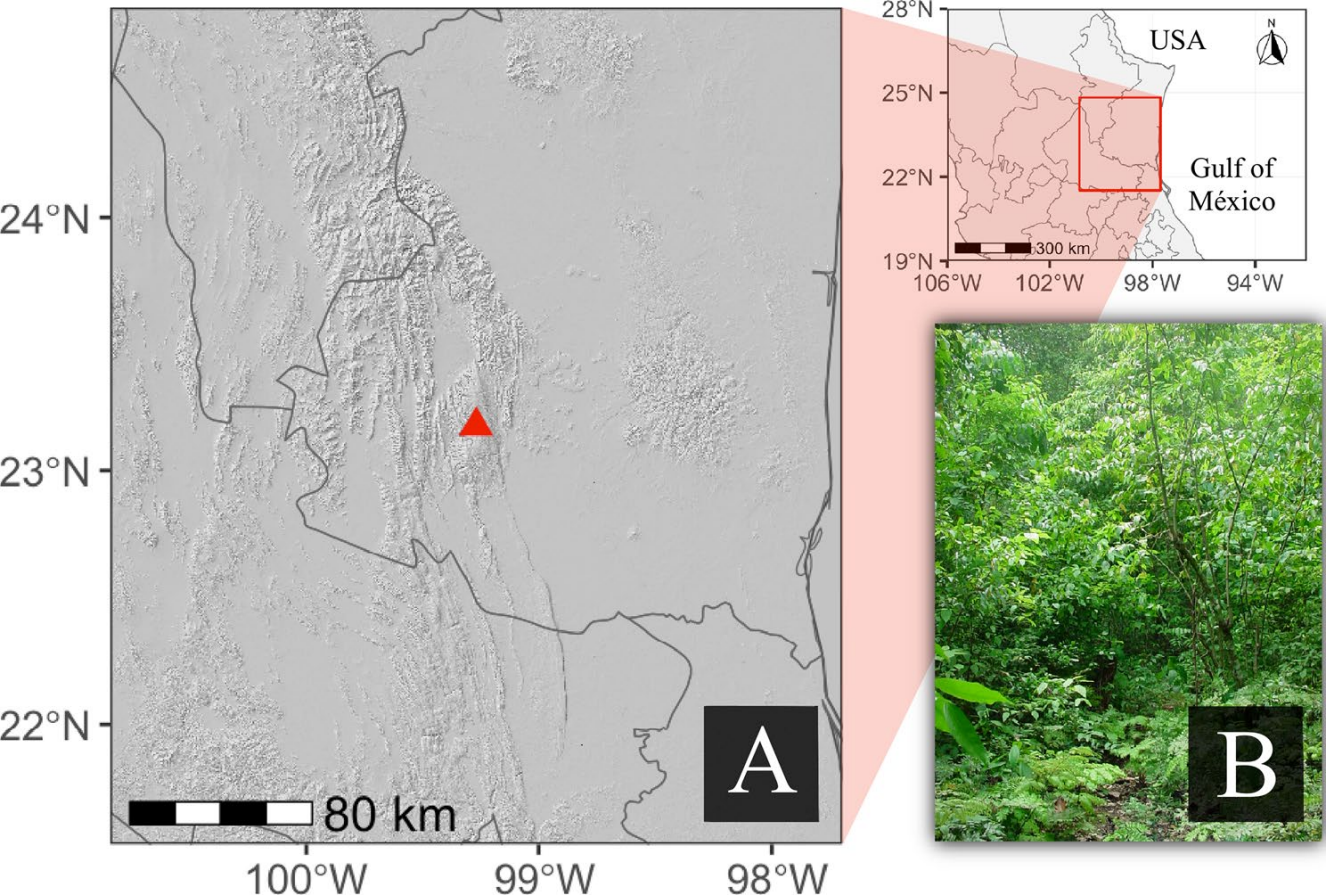
(**a**) Location of the study site within the “El cielo” biosphere reserve, Gómez Farías, Tamaulipas, Mexico, indicated by red triangle. (**b**) Tropical subdeciduous forest at the study site, the dominant vegetation type in the area

Monitoring was conducted in a five-hectare plot within the “El Cielo” Biosphere Reserve in the municipality of Gómez Farías, Tamaulipas, Mexico (Fig. 1a). We carried out 24 field visits between July 2000 and February 2004. The dominant vegetation corresponds to tropical sub-deciduous forest (Fig. 1b). The climate is warm subhumid, with a mean annual temperature of 21 °C and mean annual precipitation of 1,245 mm (Gómez Farias meteorological station [20]).

During each visit, we located and marked all crevices occupied by *X. platyceps* to assess site fidelity and refuge characteristics. Lizards were manually captured inside their refuges and individually marked by toe-clipping. For each lizard, we recorded body mass (BM) with a spring balance (100 ± 1 g), snout–vent length (SVL) with a plastic rule (30 ± 1 cm), and body temperature (BT) with a digital thermometer (±0.1 °C) inserted into the cloaca. For each crevice, we measured structural variables (height, width, depth, distance from the ground, and angle relative to the horizontal) and microclimatic variables (internal crevice temperature [IT] and external ambient temperature [ET]).

Given the structural complexity and proximity of available crevices, we did not calculate home-range extent using traditional metrics such as Minimum Convex Polygon or Kernel Density Estimation [2, 5]. Furthermore, the temporal scale of the study aligns with this decision. Instead, we interpreted the repeated use of the same refuge across multiple visits throughout the 3.5-year study period as evidence of strong site fidelity and minimum spatial displacement. This was considered a proxy for reduced home range size in the context of a crevice-specialised species with limited movement capacity.

**Fig. 2:**
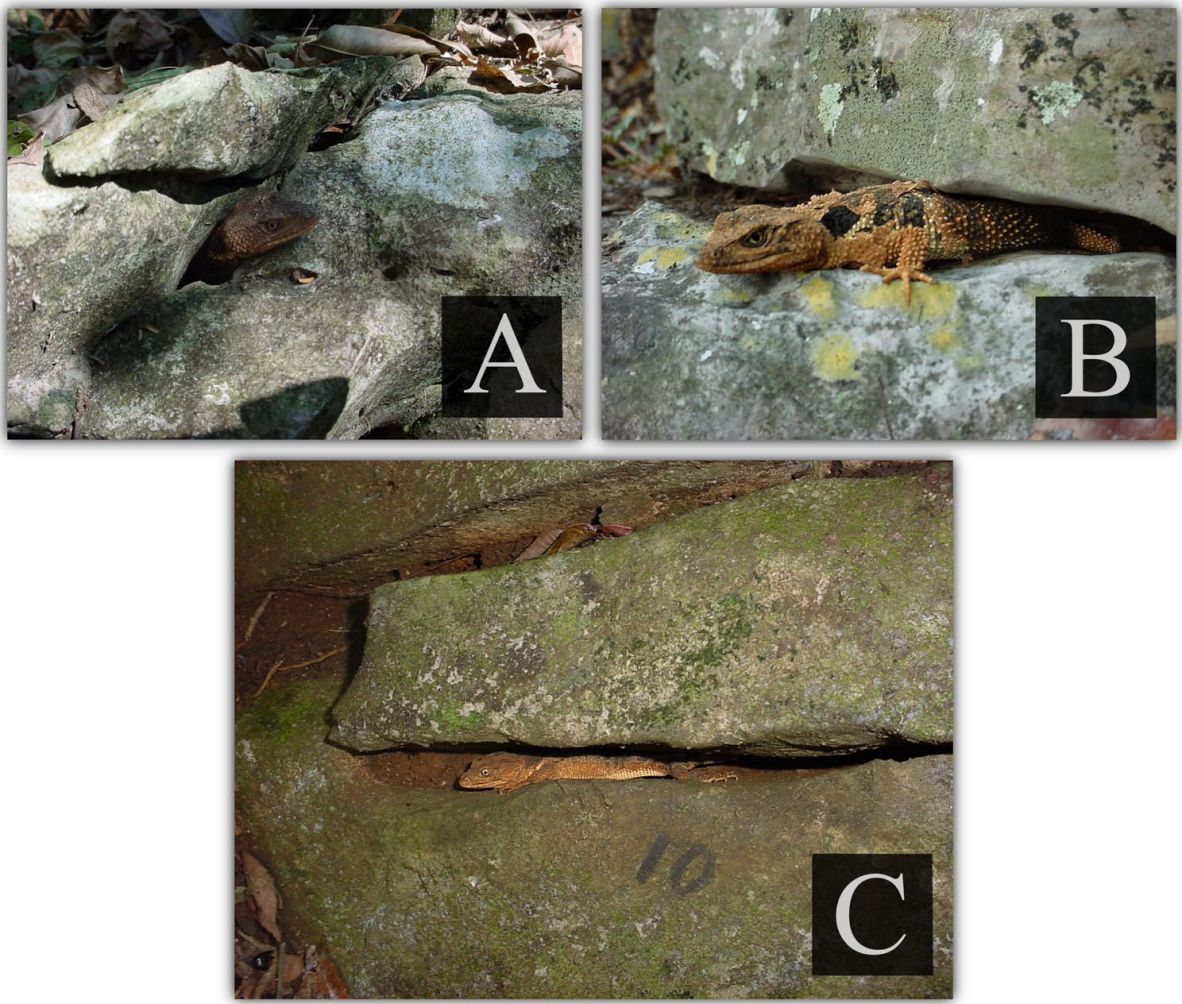
(**A–b**) adult individuals of *Xenosaurus platyceps* visible partially inside their crevices during daytime, typically exposing only the head or the anterior portion of the body. (**c**) Adult female recorded repeatedly in crevice 10, the refuge where this individual was found throughout the 43.5-month monitoring period. Photographs taken in the field using a Sony FD MAVICA camera

Importantly, throughout the entire monitoring period, no individual was ever observed outside a crevice. Surveys were conducted between 6:00 and 19:00 h. During daylight hours, individuals sometimes protruded partially from the refuge with their eyes open (Figs. 2a and b), whereas during hours without sunlight they remained completely inside with their eyes closed. This suggests a strict and consistent association with this microhabitat. In total, we monitored approximately 218 individuals, of which 14 and 23 showed consistent records of occupying the same crevice for more than one year and at least six months, respectively.

To assess whether the observed home range of *X. platyceps* deviates from expectations based on body size, we compiled estimates of minimum home range and adult body size (SVL) for other lizard species (see Additional file). Rather than conducting an exhaustive review, we based our selection on two comprehensive sources—Perry & Garland [6] and Meiri [19]—which summarise home range and morphological data across a broad range of lizard taxa. From these sources, we identified species for which both variables were reported and consulted the original references to extract the relevant data. When studies reported a range of home-range values, we consistently extracted the minimum value to ensure comparability with our minimum home-range estimate for *X. platyceps*.

We selected 100 lizard species to provide an ecologically and taxonomically broad comparative framework (Additional file). The dataset maximised variation in body size, foraging strategy, diet and phylogenetic diversity, based on available published estimates. This approach allowed us to contextualise the observed home-range value of *X. platyceps* without biasing the analysis toward any particular ecological type or taxonomic group. Thus, our dataset was designed to capture broad-scale patterns in the home range–SVL relationship rather than to be taxonomically exhaustive or to focus selectively on either the largest or the smallest reported home ranges.

Because diet type and foraging mode have been shown to influence the relationship between body size and home range [6], we included both traits in our compilation (Additional file). Following Meiri’s [19] classification criteria, we defined three categories for each trait. Diet: “Carnivorous” (consumption of animal prey), “Herbivorous” (consumption of vegetative and/or reproductive plant structures), and “Omnivorous” (roughly comparable intake of animal and plant resources). Foraging mode: “Active” (prey captured while moving), “Sit-and-wait” (prey captured while remaining stationary), and “Mixed” (use of both strategies). These categories were incorporated as fixed effects in the Bayesian models.

Although accounting for phylogenetic effects is often recommended in comparative analyses [21], we did not include phylogenetic structure for two reasons. First, our aim was to evaluate a general allometric expectation rather than to infer evolutionary patterns such as *tempo and mode*. Second, Perry and Garland [6] showed that incorporating phylogenetic structure does not improve the fit of the regression between home range size and SVL (R^2^ with phylogeny = 0.18 vs. R^2^ without = 0.29). These considerations support the use of non-phylogenetic models in this context.

We fitted a set of 10 Bayesian generalised linear mixed models using the *MCMCglmm* function from the “*MCMCglmm*” package [22] in R [23]. This modelling framework performs well under heteroscedasticity and deviations from normality because it allows explicit specification of the error variance structure, an advantage particularly valuable with relatively small sample sizes [22]. All continuous variables were log-transformed prior to analysis. We used the default priors implemented in *MCMCglmm* [22]. We assessed residual normality and homoscedasticity through visual inspection of diagnostic plots from equivalent frequentist linear models. For all models, each species (including *X. platyceps*) was represented by a single home-range value.

We fitted the model set in stepwise hierarchical framework to test specific hypotheses: (I) no relationship between minimum home range and SVL; (II) a single allometric effect of SVL; (III-V) diet affecting the intercept, slope, or their interaction with SVL; (VI-VIII) foraging mode affecting the intercept, slope, or their interaction with SVL; and (IX-X) additive effects of diet and foraging on the intercept and slope (Table 1). A full interaction between diet and foraging could not be estimated reliably owing to parameter identifiability problems.

**Table 1:** Candidate models relating minimum home range (MHR) to body size (SVL), diet (**d**) and foraging mode (FM).

Model	Structure (fixed effects)	Statistical components	Biological hypothesis
I	~1	β₀ shared (no SVL, no factors)	No scale relationship or ecological effects on MHR.
II	~ log(SVL)	β₀ shared; β₁ shared	A single scale MHR–SVL relationship applies to all species.
III	~ log(SVL) + D	β₀ varies among diets; β₁ shared	Diet alters average MHR, but the MHR–SVL scale relationship is constant across diets.
IV	~ log(SVL) + log(SVL):D	β₀ shared; β₁ varies among diets	The MHR–SVL scale relationship depends on diet, while mean MHR remains constant.
V	~ log(SVL) * D	β₀ and β₁ both vary among diets	Both average MHR and the MHR–SVL scale relationship differ among diet categories.
VI	~ log(SVL) + FM	β₀ varies among foraging modes; β₁ shared	Foraging mode shifts average MHR, but the MHR–SVL scale relationship is constant.
VII	~ log(SVL) + log(SVL):FM	β₀ shared; β₁ varies among foraging modes	The MHR–SVL scale relationship depends on foraging mode, while mean MHR remains constant.
VIII	~ log(SVL) * FM	β₀ and β₁ both vary among foraging modes	Both average MHR and the MHR–SVL scale relationship differ among foraging strategies.
IX	~ log(SVL) + D + FM	β₀ varies additively with diet and foraging; β₁ shared	Diet and foraging independently shift average MHR; all species share the same MHR–SVL scale relationship.
X	~ log(SVL) + log(SVL):D + log(SVL):FM	β₀ shared; β₁ varies additively with diet and foraging	The MHR–SVL scale relationship changes with diet and with foraging mode, but average MHR is shared.

Model convergence was assessed by visual inspection of trace plots and autocorrelation functions, together with the effective sample size (ESS). Models were deemed convergent when ESS > 1000 for both fixed effects and the variance–covariance matrix [22].

We performed model selection based on the Deviance Information Criterion (DIC [24]), which evaluates the fit of Bayesian models by combining model goodness of fit with a penalty for complexity [25]. We considered the model with the lowest DIC to be the best-fitting model. Differences smaller than 5 units were interpreted as indicating competing models, while differences greater than 5 units indicated substantial support for one model over the other [24].

Using the posterior estimates from the regression models, we calculated the expected home range value for *X. platyceps* based on its body size and compared it with the observed value. This allowed us to evaluate whether *X. platyceps* deviates from expectations based on general allometric trends.

To evaluate thermal variation among body, internal crevice, and external ambient temperatures, we fitted two additional Bayesian models using *MCMCglmm*. The first model included only an intercept, treating the temperature observations as a pooled response. The second model added temperature type (BT, IT, ET) as a fixed effect. As above, model selection was conducted using DIC.

To assess thermal behaviour of *X. platyceps*, we applied the criterion of Huey and Slatkin [26]. Slopes close to zero in regressions between body and environmental temperatures (IT and ET) indicate active thermoregulation, whereas slopes close to one suggest thermoconformity. Additionally, if the correlation between body and ambient temperature is higher than the correlation with crevice temperature, a heliothermic trend is assumed. Otherwise, a thigmothermic trend is inferred.

To evaluate these relationships, we fitted separate *MCMCglmm* models using body temperature as response variable and each of the following as single predictors: internal crevice temperature, external ambient temperature, body mass, and SVL. Model selection was again based on DIC.

## Results

A total of 218 individuals and 314 crevices used by *X. platyceps* were identified and marked during the study. Based on the adult size threshold (SVL ≥ 100 mm [27]), 82 individuals were classified as juveniles, and the remaining 136 as adults. From these, 19 adults and 4 juveniles (~10% of all individuals) showed a minimum residence time of at least six months in the same crevice (Table 2).

**Table 2:** Site-fidelity records for *X. platyceps* (2000–2004), including crevice id, dates, duration, temperatures at first capture, morphometrics, crevice dimensions, and estimated minimum home range (m^2^).

Mark		Date		Time		Temperature (°C)			Morphometric		Crevice		Home range
Individual	Refuge	From	To	Days	Months	Body	Crevice	Environment	SVL (mm)	Mass (g)	Length (mm)	Width (mm)	(m^2^)
**6**	**10**	**30-jul-00**	**25-feb-04**	**1305**	**43.5**	**20.33**	**20.74**	**21.38**	**120**	**29.5**	**21**	**7**	**0.014**
23	36	28-oct-00	25-feb-04	1215	40.5	22.2	21.4	22.3	103	18.5	42	35	0.147
50	7	26-feb-01	18-jun-03	842	28.06	22.75	22.96	24.76	120	31.5	25	22	0.055
57	102	26-feb-01	18-jun-03	842	28.06	24.2	23.4	26.05	103	20.75	47	19	0.089
128	210	24-nov-01	25-feb-04	823	27.43	20.9	21.4	12.4	114	29.5	16	23	0.036
72	132	30-abr-01	18-jun-03	779	25.96	23.4	22.8	24.6	120	30	23.5	35	0.082
126	208	24-nov-01	20-nov-03	726	24.2	18.35	17.65	19	107	20	13.7	23	0.031
104	191	29-oct-01	15-sep-03	686	22.86	21.5	21.5	21.7	114	28	NA	NA	NA
19	138	30-abr-01	04-feb-03	645	21.5	23.7	22.9	23.4	114	28.75	37	4.27	0.015
97	150	25-sep-01	18-jun-03	631	21.03	26	25.5	27	103	20.5	28	28	0.078
100	159	29-oct-01	18-jun-03	597	19.9	NA	23.3	22.9	89	12	4.5	21	0.009
90	72	26-feb-02	18-jun-03	477	15.9	18.9	18.25	18.92	107	24.25	29	23	0.066
70	105	26-feb-01	12-jun-02	471	15.7	25.3	23.5	26	118	31	10.5	21.8	0.022
30	52	27-nov-00	27-dec-01	395	13.16	19.2	18.1	19.3	98	16	33	14	0.046
51	45	26-feb-01	27-dec-01	304	10.13	21.8	22.35	25.65	105	22.75	7.5	17.7	0.013
49	91	26-feb-01	24-nov-01	271	9.03	NA	20.5	21.2	72	6.75	7.7	16.5	0.012
82	133	25-sep-01	12-jun-02	260	8.66	23.1	22.7	21.6	106	26.25	52.7	34.5	0.181
707	151	25-sep-01	12-jun-02	260	8.66	23.1	22.7	21.9	120	32.5	49	17.5	0.085
56	101	26-feb-01	29-oct-01	245	8.16	25	23.5	27.7	112	24.5	32	15.2	0.048
101	186	29-oct-01	12-jun-02	226	7.53	NA	NA	NA	69	6	17	25.2	0.042
23	116	26-mar-01	29-oct-01	217	7.23	19.4	19	21.7	102	20.25	20.7	36.3	0.075
133	213	27-dec-01	31-jul-02	216	7.2	15.9	15	17.6	113	30	45	24	0.108
115	262	31-jul-02	04-feb-03	188	6.26	NA	NA	NA	75	6.75	NA	NA	NA

These individuals remained in the same refuge for an average of 565 days (SD = 321.1, median = 477; *n* = 23), with residence times ranging from 188 to 1305 days. Their mean snout–vent length (SVL) was 104.5 mm (SD = 15.1, range = 69–120). Minimum home range was estimated using crevice internal area as a proxy, an approach appropriate for extreme crevice specialists. The mean estimated value was 0.061 m^2^ (SD = 0.05, range = 0.01–0.18).

On July 30, 2000, crevice 10 was occupied by an adult female with an SVL of 120 mm and a body mass of 29.5 g (Fig. 2c). Based on crevice dimensions (Table 2), the estimated home-range proxy was 0.014 m^2^. This individual showed strong site fidelity. It was found in the same crevice during every visit over the entire 43.5-month study period (Table 2). On January 28, 2001, it was found sharing the crevice with two juveniles, and on February 4, 2003, with an adult male.

Model selection indicated that the additive model with SVL plus diet and foraging mode as independent main effects was the best-fitting model (DIC = 311.7, ΔDIC = 0; Table 3). Diet categories distinguish carnivorous, herbivorous and omnivorous species, while foraging modes distinguish active, sit-and-wait and mixed species (see Methods). The second-best model was the additive model with SVL and diet (DIC = 312.6, ΔDIC = 0.9). Except for the null model and the full interaction model for foraging, all remaining models showed similar support (ΔDIC < 5; Table 3). Because multiple models were competitive under the DIC criterion, we report the best-fitting model.

**Table 3:** DIC and ΔDIC (relative to the underlined best-fitting model) for Bayesian GLMMs of MHR as a function of SVL, diet and foraging mode.

Model	Fixed-effects structure	DIC	ΔDIC
IX	SVL + D + FM	311.68	0
III	SVL + D	312.57	0.89
X	SVL + SVL:D + SVL:FM	312.81	1.13
IV	SVL + SVL:D	313.21	1.53
V	SVL * D	314.19	2.51
II	SVL	315.95	4.26
VI	SVL + FM	315.95	4.2
VII	SVL + SVL:FM	316.15	4.47
VII	SVL * FM	319.6	7.92
I	~1	321.01	9.33

**Fig. 3:**
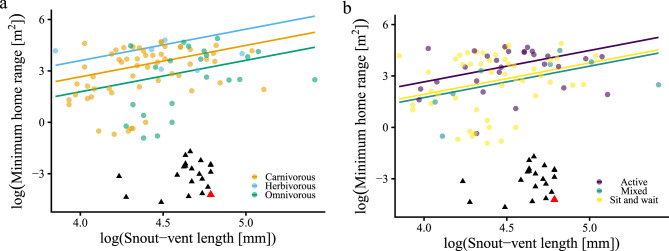
Relationships from the best-fitting model between log-transformed snout–vent length and log-transformed minimum home range across 100 lizard species. (**a**) Diet. (**b**) Foraging mode. Black triangles represent *Xenosaurus platyceps* observations; the long-term resident individual (43.5 months in the same crevice) is highlighted in red. Individual data for *X. platyceps* were included only for visualization and were not used in the comparative model

Under the best-fitting model, minimum home range increased with SVL (β₁ 95% CI = 0.53–2.86, *p*MCMC = 0.001), and diet and foraging categories differed primarily through shifts in the intercept (Fig. 3). Complete posterior estimates and category-specific regression equations are provided in the Additional file.

Based on the mean SVL of individuals that remained in the same refuge for more than six months, the expected minimum home range for carnivorous and sit-and-wait species with adult SVL comparable to *X. platyceps* were 44.9 m^2^ and 21.9 m^2^, respectively, which were 44.8 m^2^ and 21.8 m^2^ larger than the observed mean (Fig. 3). In relative terms, this represents a home range over two orders of magnitude smaller than predicted from allometric expectations.

**Fig. 4:**
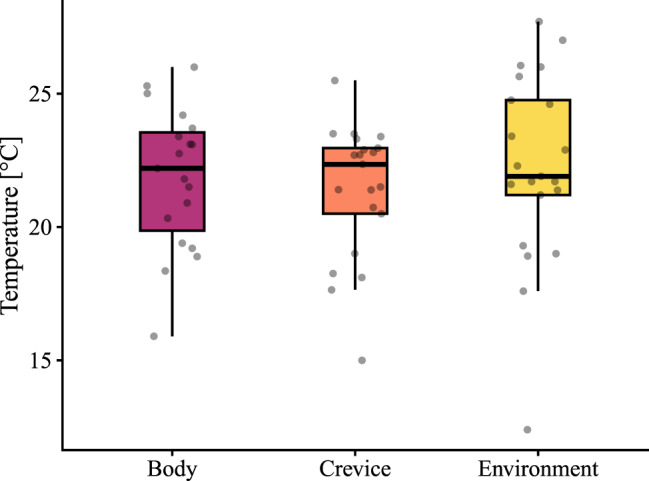
Boxplots showing the distribution of body temperature (*n* = 19), internal crevice temperature (*n* = 21), and external crevice temperature (*n* = 21) recorded in refuges used by site-fidelity individuals of *X. platyceps*. Points represent individual measurements

Mean body temperature, internal crevice temperature, and external ambient temperature recorded for site-fidelity individuals were 21.8 °C (range = 15.9–26.0; SD = 2.7; *n* = 19), 21.4 °C (range = 15.0–25.5; SD = 2.5; *n* = 21), and 22.2 °C (range = 12.4–27.7; SD = 3.6; *n* = 21), respectively (Fig. 4). Model selection showed that the model assuming no differences among the three temperatures was the best-fitting model (DIC = −59.1). The model including temperature category fit worse (DIC = −55.5; ΔDIC = 3.6).

Model selection for the predictors of body temperature indicated that the model including internal crevice temperature as a predictor was the best-fitting model (DIC = −76.5, ΔDIC = 0). This model was strongly supported over the one using external ambient temperature (DIC = −30.7, ΔDIC = 45.8), the null model (DIC = −21.63, ΔDIC = 54.9), and SVL model (DIC = −20.1, ΔDIC = 56.4). The body-mass model (DIC = 20, ΔDIC = 96.5) showed the worst fit among all evaluated models.

**Fig. 5:**
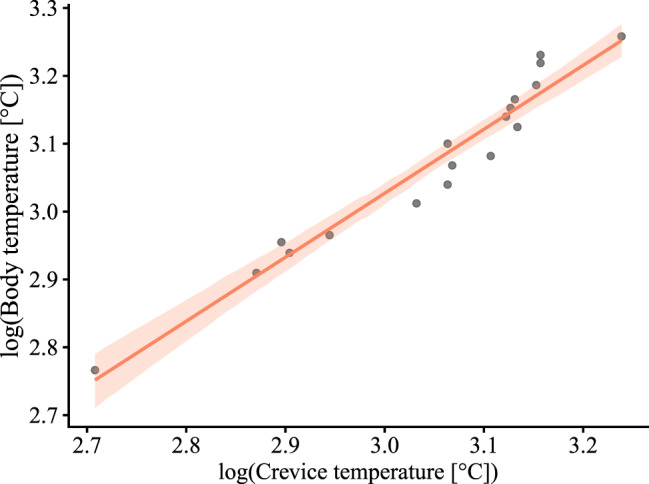
Relationship between log-transformed body temperature and log-transformed crevice temperature in X. platyceps. The regression line and 95% credible interval represent the posterior estimates from the best-fitting Bayesian model selected using DIC

The relationship between body and crevice temperature was (Fig. 5):

log(Body temperature) = 0.19 + 0.94 × log(crevice temperature)

with posterior intervals β₁ 95% CI = 0.82 to 1.04, pMCMC < 0.001; β₀ 95% CI = −0.14 to 0.53, pMCMC = 0.23.

## Discussion

Small home ranges are commonly associated with early developmental stages or with specific behavioural conditions. For example, home ranges smaller than 1 m^2^ have been reported in juveniles of species such as *Anolis aeneus*, *A. lineatopus*, and *Sceloporus minor* [6, 19, 28]. Likewise, *Amblyrhynchus cristatus* can markedly reduce its home range during territorial disputes (from 38.8 m^2^ to 1.2 m^2^ [29]). These examples suggest that extremely small home ranges are typically associated with early life stages or specific behavioural contexts.

In contrast, with the exception of 4 juveniles of 23 individuals, the small home ranges observed in *X. platyceps* were recorded in fully grown adults. Moreover, these individuals maintained stable home ranges for at least six months, in some cases, for more than three years. Among them, we observed one adult female with an exceptional minimum home range (Fig. 2c). This pattern suggests that *X. platyceps* may represent a case of prolonged spatial restriction not driven by ontogenetic development or transient behavioural patterns.

Beyond ontogeny and behavioural context, body size offers an additional comparative framework. Home ranges < 1 m^2^ in *Anolis aeneus, A. lineatopus* and *Sceloporus minor* occur in species that are substantially smaller than *X. platyceps* (about one-third to one-half its adult size [19]). This pattern also emerges when examining species of similar body size and habitat use. For instance, *Laudakia tuberculata,* a rock-dwelling lizard with a comparable body size to *X. platyceps* (~120 mm SVL [30]), exhibits a substantially larger minimum home range (45.7 m^2^ vs. 0.061 m^2^ [19]). These contrasts indicate that the extremely small home ranges observed in *X. platyceps* cannot be attributed to body size.

This observation is reinforced by our comparative allometric analysis. The general patterns recovered by these models match those previously documented for lizards: home range increases with body size, and trophic and foraging traits introduce predictable shifts in spatial use [6, 9]. However, *X. platyceps* departs sharply from these trends (Fig. 3). This deviation may indicate that habitat specialisation can override classical allometric expectations, constraining movement and decoupling home-range extent from typical predictors.

We also identified three important caveats in our analysis. First, several alternative models were competitive (Table 2). This indicated limited ability to distinguish the specific contributions of diet and foraging within the present dataset. Moreover, general ecological traits may also perform poorly as predictors in habitat-specialist lizards constrained by crevice microhabitats, such as *X. platyceps*. This suggests that other ecological factors may play a stronger role in such specialists. Thus, inferences about the individual effects of diet and foraging should be interpreted with caution.

Second, home-range measurements used in comparative analyses may introduce methodological bias. Multiple approaches for quantifying home-range extent have been employed across studies, depending on the focal taxon and the methodological preferences or constraints of each research group [2, 5]. This issue has been acknowledged in previous comparative analyses of home range [6] and in other ecological questions [31, 32]. We consider that using the internal area of the crevice as a measure of minimal home range more accurately reflects the natural conditions of extreme habitat specialists, such as *X. platyceps*. This approach makes it possible to incorporate extreme habitat specialists into a comparative analytical framework. However, we also recognise that our analyses represent an approximation that may help improve our understanding of species with secretive habits.

Third, the phylogenetic structure of our comparative dataset may influence our results. Although the phylogenetic models of Perry & Garland [6] did not outperform non-phylogenetic ones, this may reflect taxon sampling rather than a true absence of phylogenetic signal. Our dataset included species from highly divergent lineages, such as lacertids and iguanids. Comparative analyses incorporate phylogenetic distance as a covariance matrix, and therefore depend on branch lengths [33]. Moreover, weak evolutionary signals can be difficult to detect even when phylogenetic distances are short [34]. Therefore, the large biological disparity represented by long phylogenetic distances may mask subtle phylogenetic effects. Future efforts focused on assembling minimum home-range datasets for a genus or small lizard family could help clarify the extent and drivers of these patterns.

Site fidelity was also high, with several individuals remaining in the same refuge for extended periods, including one long-term resident that stayed in the same crevice for more than three years (Fig. 2c). Although site fidelity has been documented in other reptile species [7, 35, 36], we are not aware of reports describing such prolonged use of a single refuge. Such extreme fidelity could reflect the stability of these crevice conditions (e.g., low environmental variation, high food availability, few neighbours) that have been suggested to promote both site fidelity and reduced home ranges [37]. This level of spatial restriction may also entail ecological or evolutionary costs and benefits that warrant further investigation, particularly under scenarios of environmental change or habitat loss.

One factor that may help explain both the reduced home range and high site fidelity is the thermal ecology of *X. platyceps*. Model selection indicated that body, ambient, and refuge temperatures were similar, suggesting a thermally stable environment [12]. Furthermore, the strong relationship between body temperature and internal crevice temperature (best-fitting model) is consistent with thermoconformity and thigmothermy in *X. platyceps* [26]. Similar patterns have been reported for other species in the genus [16–18]. This suggests that *X. platyceps* may not need to move actively to maintain thermal balance, as crevices likely provide relatively stable conditions. This thermal buffering could help explain the extended use of a single refuge.

In addition to thermal behaviour, *Xenosaurus* lizards exhibit morphological traits that may promote reduced home ranges and strong site fidelity. For instance, *X. platyceps* presents a dorsoventrally flattened body and limbs positioned laterally and perpendicular to the trunk. These are traits of organisms that live in narrow crevices [38, 39]. Furthermore, *X. platyceps* possess osteoderms that may assist in anchoring themselves and remaining concealed within these confined spaces. These traits likely reduce locomotor efficiency in open areas and favour prolonged refuge use.

Diet may also play an important role in space use. The diet of *X. platyceps* is composed primarily of coleopterans and orthopterans, but it is not restricted to these groups [40]. Moreover, *Xenosaurus* individuals have been documented consuming relatively large prey items, including mammals, lizards, and snakes [41–43]. Consistent with this diverse diet, they are ambush predators that chemically detect nearby prey [40, 44]. This generalist feeding strategy likely allows individuals to exploit prey encountered near their refuges, reducing the need for extensive movement [45].

Reproductive behaviour may also influence spatial use. In several lizard species, females occupy smaller home ranges than males [6]. This suggests that males may use a larger spatial area to locate mates, whereas females may benefit from remaining in a stable refuge that facilitates reproduction and offspring protection. If similar patterns occur in *Xenosaurus*, reproductive roles may help reinforce prolonged refuge fidelity.

Finally, microhabitat use may further limit movement. Observations in captivity and in the field indicated that individuals defecate in latrines located near the edges of crevices (on average 11 cm from the crevice entrance; pers. obs.). This indicates that they likely do not move far even for essential activities. Such behaviour may represent an additional mechanism promoting restricted spatial use.

It is important to note that not all crevices showed the same pattern. In some refuges we observed frequent occupancy by different individuals, suggesting turnover rather than long-term fidelity. This indicates that the extreme case documented here represents one end of a broader behavioural continuum within the species. A similar, though less extreme, pattern has been reported for another population of *X. platyceps* inhabiting a temperate environment with cooler and more heterogeneous temperatures [46]. This suggests that site fidelity may be consistent across populations, although variation in thermal regimes could modulate its magnitude.

Knowledge about the home-range dynamics of many secretive vertebrates remains limited. Species with lifestyles similar to that of *Xenosaurus* may experience comparable ecological constraints. Habitat fragmentation has been shown to negatively affect space use in lizards [47], suggesting that extreme habitat specialists could be particularly sensitive to local disturbances. Although behavioural plasticity may buffer short-term impacts of environmental change and habitat loss, a restricted ecological niche may limit such buffering [48]. These considerations highlight the potential value of incorporating site fidelity and restricted spatial use as vulnerability indicators in conservation assessments at both national (e.g., NOM-059) and international levels (e.g., IUCN Red List).

## Conclusions

Our long-term field observations reveal exceptional spatial confinement and high site fidelity in the crevice-dwelling lizard *X. platyceps*. Thermal measurements indicate a thermally stable microenvironment and a pattern consistent with thermoconformity and thigmothermy, offering a mechanistic explanation for reduced movement. Furthermore, comparative Bayesian analyses confirm a positive allometric relationship between body size and minimum home range, with additive shifts by diet and foraging mode. However, these general predictors markedly overestimate the space use of *X. platyceps*, demonstrating that strong microhabitat specialisation can decouple spatial behaviour from classical body-size expectations.

Future research should explore whether similar patterns occur in other crevice specialists. Comparisons among populations exposed to different environmental regimes would also help clarify how temperature and habitat structure shape space use. In addition, incorporating sex and age structure into movement analyses may reveal demographic differences in spatial behaviour. Finally, applying tools designed to track movement in physically complex environments could improve the precision of home-range estimates in cryptic or habitat specialists.

## Electronic supplementary material

Below is the link to the electronic supplementary material.

Supplementary Material 1

## Data Availability

No datasets were generated or analysed during the current study.
